# Machine Learning Consensus Clustering Approach for Patients with Lactic Acidosis in Intensive Care Units

**DOI:** 10.3390/jpm11111132

**Published:** 2021-11-02

**Authors:** Pattharawin Pattharanitima, Charat Thongprayoon, Tananchai Petnak, Narat Srivali, Guido Gembillo, Wisit Kaewput, Supavit Chesdachai, Saraschandra Vallabhajosyula, Oisin A. O’Corragain, Michael A. Mao, Vesna D. Garovic, Fawad Qureshi, John J. Dillon, Wisit Cheungpasitporn

**Affiliations:** 1 Department of Internal Medicine, Faculty of Medicine, Thammasat University, Pathum Thani 12121, Thailand; 2 Division of Nephrology and Hypertension, Department of Medicine, Mayo Clinic, Rochester, MN 55905, USA; garovic.Vesna@mayo.edu (V.D.G.); Qureshi.Fawad@mayo.edu (F.Q.); dillon.John@mayo.edu (J.J.D.); 3 Division of Pulmonary and Pulmonary Critical Care Medicine, Faculty of Medicine, Ramathibodi Hospital, Mahidol University, Bangkok 10400, Thailand; petnak@yahoo.com; 4 Division of Pulmonary Medicine, St. Agnes Hosipital, Baltimore, MD 21229, USA; nsrivali@gmail.com; 5 Unit of Nephrology and Dialysis, Department of Clinical and Experimental Medicine, University of Messina, 98125 Messina, Italy; guidogembillo@live.it; 6 Department of Military and Community Medicine, Phramongkutklao College of Medicine, Bangkok 10400, Thailand; wisitnephro@gmail.com; 7 Division of Infectious Disease, Department of Medicine, Mayo Clinic, Rochester, MN 55905, USA; s.chesdachai@gmail.com; 8 Section of Cardiovascular Medicine, Department of Medicine, Wake Forest University School of Medicine, Winston-Salem, NC 27101, USA; svallabh@wakehealth.edu; 9 Department of Thoracic Medicine and Surgery, Temple University Hospital, Philadelphia, PA 19140, USA; 109426469@umail.ucc.ie; 10 Division of Nephrology and Hypertension, Department of Medicine, Mayo Clinic, Jacksonville, FL 32224, USA; mao.michael@mayo.edu

**Keywords:** lactic acid, lactic acidosis, lactate, hyperlactatemia, clustering, intensive care unit, machine learning, artificial intelligence, critical care, critical care medicine, nephrology, precision medicine, personalized medicine, individualized medicine

## Abstract

Background: Lactic acidosis is a heterogeneous condition with multiple underlying causes and associated outcomes. The use of multi-dimensional patient data to subtype lactic acidosis can personalize patient care. Machine learning consensus clustering may identify lactic acidosis subgroups with unique clinical profiles and outcomes. Methods: We used the Medical Information Mart for Intensive Care III database to abstract electronic medical record data from patients admitted to intensive care units (ICU) in a tertiary care hospital in the United States. We included patients who developed lactic acidosis (defined as serum lactate ≥ 4 mmol/L) within 48 h of ICU admission. We performed consensus clustering analysis based on patient characteristics, comorbidities, vital signs, organ supports, and laboratory data to identify clinically distinct lactic acidosis subgroups. We calculated standardized mean differences to show key subgroup features. We compared outcomes among subgroups. Results: We identified 1919 patients with lactic acidosis. The algorithm revealed three best unique lactic acidosis subgroups based on patient variables. Cluster 1 (*n* = 554) was characterized by old age, elective admission to cardiac surgery ICU, vasopressor use, mechanical ventilation use, and higher pH and serum bicarbonate. Cluster 2 (*n* = 815) was characterized by young age, admission to trauma/surgical ICU with higher blood pressure, lower comorbidity burden, lower severity index, and less vasopressor use. Cluster 3 (*n* = 550) was characterized by admission to medical ICU, history of liver disease and coagulopathy, acute kidney injury, lower blood pressure, higher comorbidity burden, higher severity index, higher serum lactate, and lower pH and serum bicarbonate. Cluster 3 had the worst outcomes, while cluster 1 had the most favorable outcomes in terms of persistent lactic acidosis and mortality. Conclusions: Consensus clustering analysis synthesized the pattern of clinical and laboratory data to reveal clinically distinct lactic acidosis subgroups with different outcomes.

## 1. Introduction

Lactic acidosis is the most common anion gap metabolic acidosis in critically ill patients [1,2]. It has been associated with adverse outcomes, including increased mortality [1,3,4,5]. Lactic acidosis in intensive care units (ICUs) typically occurs in the setting of tissue hypoperfusion, such as in shock (type A lactic acidosis), or less commonly with type B lactic acidosis (associated with liver failure, malignancies, or certain drugs such as biguanides) [6,7]. While it has been consistently shown that lactic acidosis with a serum lactate value greater than 4 mmol/L is a poor survival prognostic marker for ICU patients [3,4,8,9,10,11,12], ICU patients with lactic acidosis are an extremely heterogeneous population placed in diverse clinical situations and settings, such as sepsis, trauma, or cardiac surgeries [9,10,11,13,14,15,16,17,18,19,20,21]. In addition, the effects of lactic acidosis in these various settings are diverse [9,10,11,13,14,15,16,17,18,19,20,21].

With the advancement of electronic medical records (EMR) and artificial intelligence, machine learning (ML) algorithms have become more widely utilized in individualized medicine to assist clinical decision-making [22,23]. Consensus clustering is an unsupervised ML technique that is utilized to identify similarities and differences among various data variables, and then assign them into meaningful clusters [24,25]. Recent studies have demonstrated that ML consensus clustering approach can distinguish meaningful disease subtypes that forecast unique clinical outcomes [26,27]. Given the heterogeneity of patients with lactic acidosis on ICU admission [9,10,11,13,14,15,16,17,18,19,20,21], ML consensus clustering approach may help clinicians better identify different phenotypes of critically ill patients with lactic acidosis in order to apply individualized strategies to improve outcomes.

In this study, we aimed to identify clinically meaningful clusters of critically ill patients with lactic acidosis on ICU admission using an unsupervised ML clustering approach. We then assessed these distinct clusters’ individual outcomes.

## 2. Methods

### 2.1. Patient Population

We used the Medical Information Mart for Intensive Care III (MIMIC III) database to identify critically ill adult patients (aged 18 years or older) with lactic acidosis at ICU admission. MIMIC III database is a publicly available critical care database of patients from a large, single-center tertiary care hospital from 2001 to 2012 [28]. Lactic acidosis was defined as the first serum lactate measured within 48 h of ICU admission of ≥4.0 mmol/L. Patients were excluded if they did not have a serum lactate measurement within 48 h of ICU admission or if they were admitted in ICU for ≤24 h. We included only the first ICU admission if patients had multiple ICU admissions. The Mayo Clinic Institutional Review Board approved this study (IRB number 21-009222) and waived the need for informed consent due to the use of publicly and de-identified database.

### 2.2. Data Collection

We abstracted EMR data on patient characteristics, comorbidities, vital signs, organ supports, and laboratory results to identify clinically distinct lactic acidosis clusters. As our goal was to cluster lactic acidosis patients based on available data at the time of ICU admission, we only used data that was present within 48 h of ICU admission for clustering analysis. We selected the first vital sign or laboratory value within the 48-h time frame when there were multiple values. We excluded laboratory results with over 10% missing data. Otherwise, missing data were multiple imputed using the Random Forest method before inclusion in cluster analysis.

We defined comorbidities using the Elixhauser Comorbidity Software, which identifies comorbidities by grouping International Classification of Disease Clinical Modification codes [29]. We calculated comorbidity burden using Charlson Comorbidity Score. [30] We calculated Severity index for the acute illness using Simplified Acute Physiology Score (SAPS) II [31]. We estimated glomerular filtration rate (GFR) using the Chronic Kidney Disease Epidemiology Collaboration (CKD-EPI) equation [32]. We defined acute kidney injury (AKI) as per the Kidney Disease Improving Global Outcome (KDIGO) guideline’ serum creatinine and urine output criteria [33]. For serum creatinine criteria, we used the lowest creatinine seven days prior to ICU admission as the baseline and compared it with the highest creatinine within 48 h of ICU admission. Patients had AKI if there was an increase in serum creatinine of ≥0.3 mg/dL or 1.5 times baseline. For urine output criteria, we split urine output within 48 h after ICU admission into 6-h time periods. Patients had AKI if the total urine output in any 6-h time interval was below the limit of 3 mL/kg. Outcomes of interest included persistent lactic acidosis (defined as all subsequent serum lactate values after initial elevated value within 48 h of its occurrence were ≥4 mmol/L), hospital mortality, and 90-day mortality after ICU admission.

### 2.3. Cluster Analysis

We applied an unsupervised ML approach to consensus clustering in order to identify clinical phenotypes of ICU patients with lactic acidosis [34]. We used a pre-specified subsampling parameter of 80% with 100 iterations and assigned the number of potential clusters (k) to range from 2 to 10 in order to avoid producing an excessive number of clusters that would not be clinically useful. The optimal number of clusters was determined by examining the consensus matrix (CM) heat map, cumulative distribution function (CDF), cluster-consensus plots in the within-cluster consensus scores, and the proportion of ambiguously clustered pairs (PAC) [35,36]. The within-cluster consensus score, ranging between 0 and 1, is defined as the average consensus value for all pairs of individuals belonging to the same cluster [36]. A value closer to one indicates better cluster stability [36]. PAC, ranging between 0 and 1, is calculated as the proportion of all sample pairs with consensus values falling within the predetermined boundaries [35]. A value closer to zero indicates better cluster stability [35]. We calculated the PAC utilizing two criteria: (1) the strict criteria consisting of a predetermined boundary of (0, 1), where a pair of individuals who had consensus value greater than 0 or less than 1 was considered ambiguously clustered, and (2) the relaxed criteria consisting of a predetermined boundary of (0.1, 0.9), where a pair of individuals who had consensus value greater than 0.1 or less than 0.9 was considered ambiguously clustered [35]. This study’s detailed consensus clustering algorithms are provided in the Online Supplementary.

### 2.4. Statistical Analysis

After we identified the clusters of lactic acidosis patients, we performed analyses to test the differences among the clusters. First, we compared patient characteristics among the clusters using the analysis of variance (ANOVA) test for continuous variables and Chi-squared test for categorical variables. We determined the clusters’ key features using standardized mean differences with a set cut-off of >0.3. We then compared outcomes among the identified clusters. We assessed the association of clusters with persistent lactic acidosis and hospital mortality using logistic regression. We assessed the association of clusters with 90-day mortality using Cox proportional hazard regression. We selected cluster 1 as the reference group because it was associated with the most favorable outcomes. We did not adjust for patient characteristics because these characteristics were utilized to identify clusters through unsupervised ML. We performed all analyses using R, version 4.0.3 (RStudio, Inc., Boston, MA, USA; http://www.rstudio.com/ (accessed on 15 January 2021)), with the packages of ConsensusClusterPlus (version 1.46.0) [36] for consensus clustering analysis, and the missForest package for missing data imputation [37].

## 3. Results

Out of 34,682 ICU admissions, 18,969 had serum lactate measurements within 48 h of ICU admission. Of these, 1919 had lactic acidosis with serum lactate of ≥4 mmol/L at ICU admission. The mean age was 62 ± 17, 58% were male, 81% were white. 11%, 24%, 32%, 15%, and 18% of patients were admitted to the cardiac, cardiac surgery, medical, surgical, and trauma/surgical ICU, respectively. The mean serum lactate was 6.2 ± 2.6 mmol/L.

**Figure 1:**
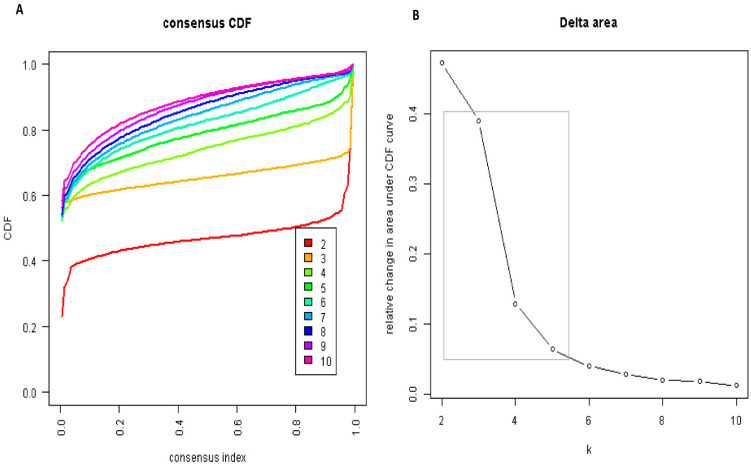
(**A**) CDF plot displaying consensus distributions for each cluster (k) for ICU patients with lactic acidosis; (**B**) Delta area plot reflecting the relative changes in the area under the CDF curve.

**Figure 2:**
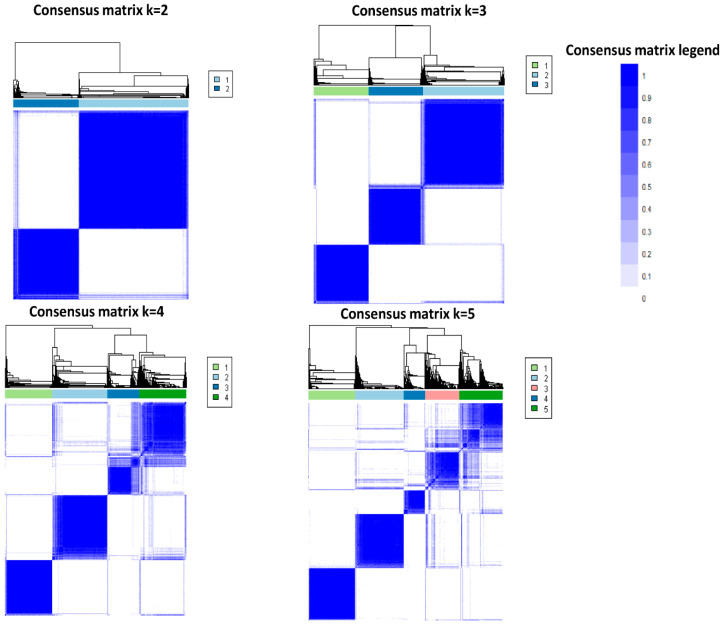
Consensus matrix heat map depicting consensus values on a white to blue color scale of each cluster.

The CDF plot displays the consensus distributions for each cluster (Figure 1A). The delta area plot shows the relative change in the area under the CDF curve (Figure 1B). The largest changes in area occurred between k = 3 and k = 5, at which point the relative increase in area became noticeably smaller. As shown in the CM heatmap (Figure 2, Supplementary Materials, Figures S1–S9), the ML algorithm identified cluster 2 and cluster 3 with clear boundaries, indicating good cluster stability over repeated iterations.

**Figure 3:**
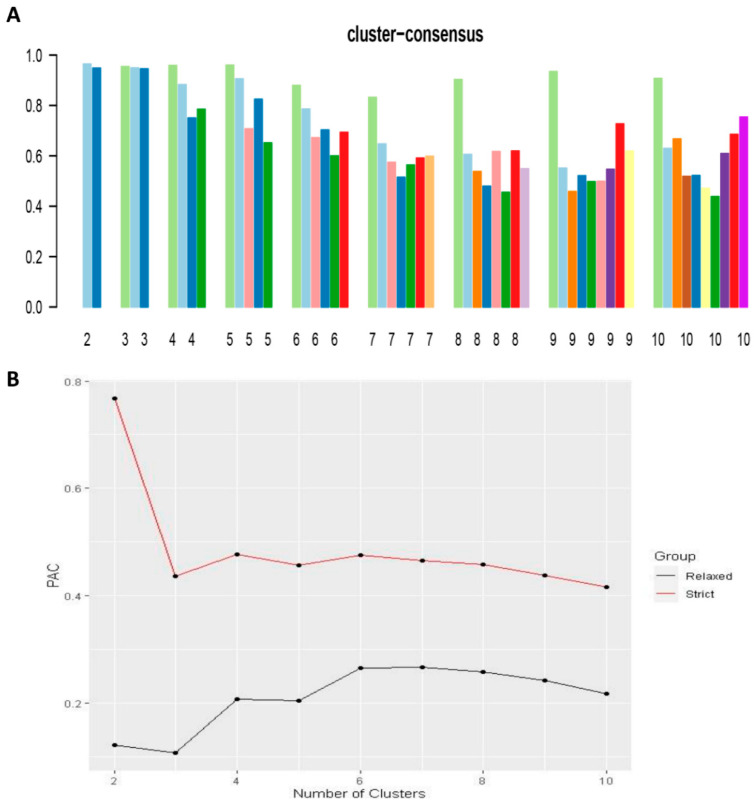
(**A**) The bar plot represents the mean consensus score for different numbers of clusters (K ranges from two to ten) for ICU patients with lactic acidosis; (**B**) The PAC values using the strict criteria (red line) with the predetermined boundary of (0, 1), and the PAC values using the relaxed criteria (black line) with the predetermined boundary of (0.1, 0.9) as the definition for ambiguously clustered pairs.

The mean cluster consensus score was comparable between a scenario of two or three clusters (Figure 3A). In addition, favorable low PACs by both strict and relaxed criteria were demonstrated for three clusters (Figure 3B). Thus, using baseline variables at hospital admission, the consensus clustering analysis identified three clusters that best represented the data pattern of our ICU patients with lactic acidosis.

There were 554 patients in cluster 1, 825 patients in cluster 2, and 550 patients in cluster 3. Table 1 shows the patient characteristics of the three identified clusters.

**Figure 4:**
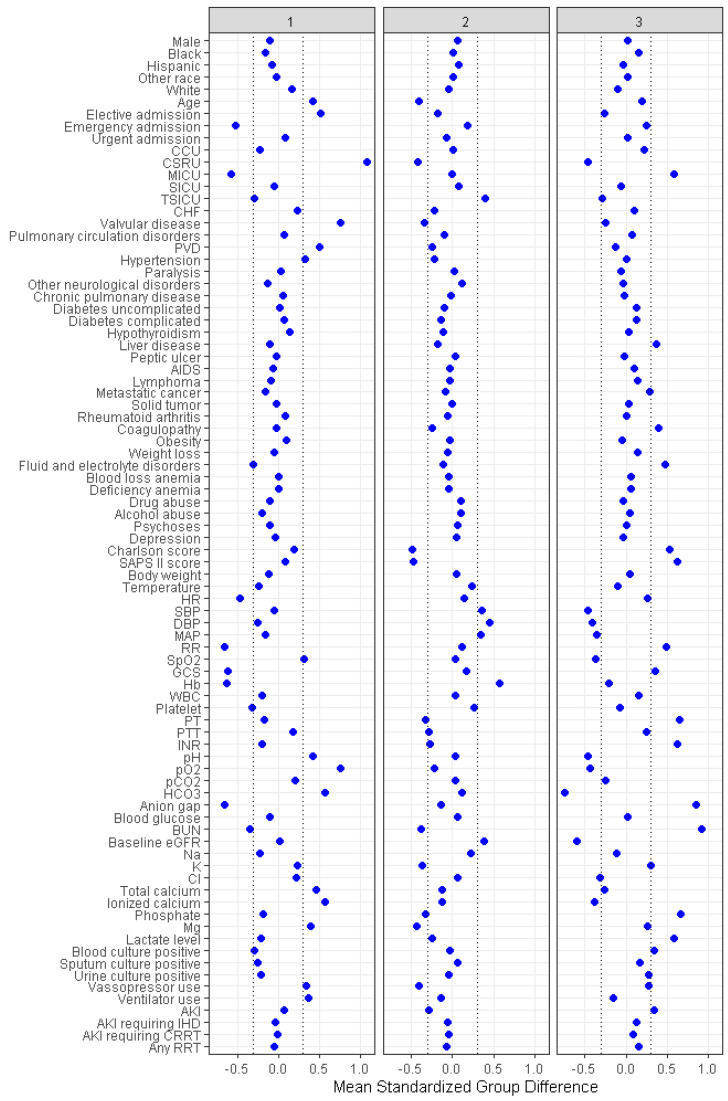
Standardized differences across the two clusters for each baseline variable for ICU patients with lactic acidosis. The *X*-axis represents the standardized differences value, and the *Y*-axis represents baseline variables. The dashed vertical lines signify the standardized differences cutoffs of <−0.3 or >0.3. Abbreviations: AIDS, acquired immune deficiency syndrome; AKI, acute kidney injury; BUN, blood urea nitrogen; CCU, coronary care unit; CHF, chronic heart failure; Cl, chloride; CRRT, continuous renal replacement therapy; CSRU, cardiac surgery recovery unit; DBP, diastolic blood pressure; eGFR, estimated glomerular filtration rate; GCS, Glasgow coma scale; Hb, hemoglobin; HR, heart rate; ICU, intensive care unit; IHD, intermittent hemodialysis; INR, international normalized ratio; K, kalium (potassium); MAP, mean arterial pressure; Mg, magnesium; MICU, medical intensive care unit; Na, natrium (sodium); pH, potential of hydrogen; pCO_2_, partial pressure of carbon dioxide; pO_2_, partial pressure of oxygen; PT, prothrombin time; PTT, partial thromboplastin time; PVD, peripheral vascular disease; RR, respiratory rate; RRT, renal replacement therapy; SAPS II Score, Simplified Acute Physiology Score II; SBD, SPO_2_, saturation of peripheral oxygen; SBP, systolic blood pressure; SICU, surgical intensive care unit; WBC, white blood cell.

The plot of standardized mean difference in Figure 4 demonstrates the key features of each cluster.

Cluster 1 was characterized by old age, elective admission to cardiac surgery ICU, and vasopressor and mechanical ventilation use. In terms of laboratory results, cluster 1 patients had higher pH, partial pressure of oxygen (pO_2_), serum bicarbonate, and magnesium, but lower hemoglobin and anion gap. Cluster 2 was characterized by young age, admission to trauma/surgical ICU with higher blood pressures, lower comorbidity burden, lower severity index, and less vasopressor use. In terms of laboratory results, cluster 2 patients had higher hemoglobin, but lower BUN, serum potassium, phosphate, and magnesium. Cluster 3 was characterized by admission to medical ICU, history of liver disease and coagulopathy, acute kidney injury, lower blood pressure, higher comorbidity burden, and higher severity index. In terms of laboratory results, cluster 3 had a higher serum lactate, anion gap, BUN, phosphate, prothrombin time (PT), international normalized ratio (INR), but lower pH, pO_2_, eGFR, serum bicarbonate, and ionized calcium.

Persistent lactic acidosis occurred in 9.2%, 9.8%, and 40% in cluster 1, cluster 2, and cluster 3, respectively (Table 2 and Figure 4).

**Figure 5:**
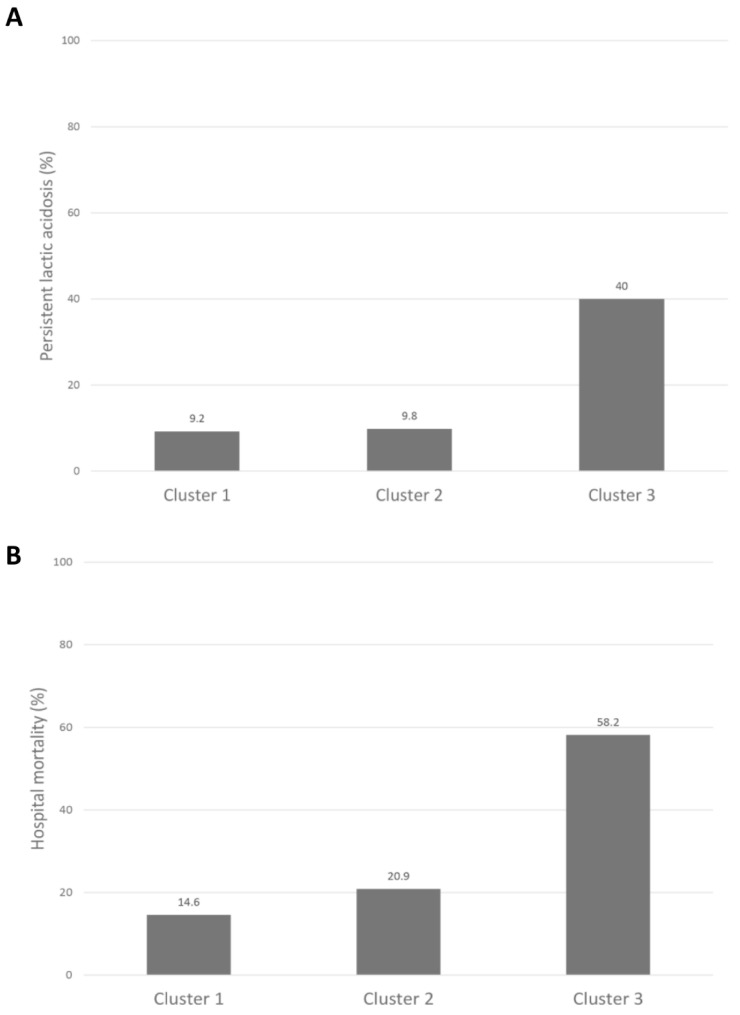
(**A**) Persistent lactic acidosis; and (**B**) hospital mortality among different clusters of ICU patients with lactic acidosis.

**Figure 6:**
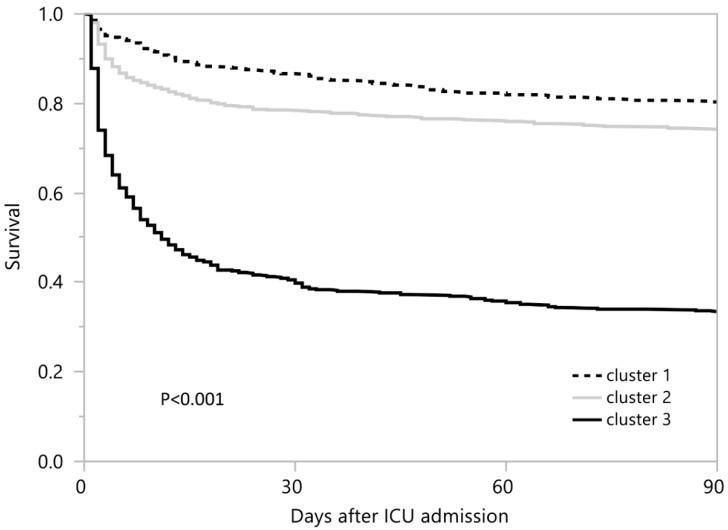
90-day mortality among different clusters of ICU patients with lactic acidosis.

Cluster 3 (OR 6.59; 95% CI 4.62–9.39), but not cluster 2, was significantly associated with persistent lactic acidosis when compared to cluster 1 (Figure 5A). Cluster 3 had the highest hospital- and 90-day mortality, followed by cluster 2, and then cluster 1. Hospital mortality was 14.6%, 20.9%, and 58.2% in cluster 1, cluster 2, and cluster 3, respectively (Table 2 and Figure 5B). Cluster 2 (OR 1.54; 95% CI 1.15–2.06) and cluster 3 (OR 8.12; 95% CI 6.08–10.86) were significantly associated with higher hospital mortality, compared to cluster 1. Ninety-day mortality was 19.9%, 25.9%, and 66.6% in cluster 1, cluster 2, and cluster 3, respectively (Table 2 and Figure 6). Cluster 2 (HR 1.38; 95% CI 1.10–1.74) and cluster 3 (HR 5.06; 95% CI 4.09–6.27) were significantly associated with higher 90-day mortality when compared to cluster 1 (Figure 6).

## 4. Discussion

ML consensus clustering algorithms offer the ability to efficiently analyze and identify unique clusters of patients with different characteristics in a large amount of data [24,25,38,39]. In this study, we identified three clinically distinct clusters of patients with lactic acidosis at time of ICU admission utilizing a ML unsupervised consensus clustering approach. The three clusters demonstrated different characteristics and were associated with unique clinical outcomes, including persistent lactic acidosis, hospital mortality, and 90-day mortality.

Cluster 1, designated as the reference cluster, consisted of patients of an older age who had underlying valvular heart disease, peripheral vascular disease, and hypertension. Of the three clusters, cluster 1 patients had the highest rate of elective ICU admission to cardiac surgery recovery Unit (CSRU) after cardiac surgery. These patients had the highest rates of vasopressor and mechanical ventilator utilization. While cluster 1 patients had the highest severity of anemia, they also had the highest level of pH, HCO_3_, and pO_2_ among all of the clusters. While cluster 1 patients had the lowest Glasgow coma scale (GCS) scores on ICU admission, they were more often admitted electively after cardiac surgery and required mechanical ventilator. This could be due to sedation. While AKI occurred in 76% of cluster 1 patients, severe AKI requiring renal replacement therapy only composed 2% of these patients. Average blood lactate level in cluster 1 was 5.7 mmol/L, and only 9.2% had persistent lactic acidosis 48 h after ICU admission. This corresponds to the literature where elevated lactate levels are frequently noted after cardiac surgery, with an incidence of 10–20% of post cardiac surgical patients [15,16,17,18,19]. The causes of lactic acidosis following cardiac surgery include both hypoxic (type A) and non-hypoxic causes (type B). Type A would include inadequate oxygen delivery during cardiopulmonary bypass (CPB), low cardiac output, severe anemia/hemodilution, and Type B would include exogenous catecholamines (epinephrine, isoproterenol, and salbutamol) [40,41]. Among these possible causes, prolonged CPB duration and intraoperative vasopressor requirements have been shown to be strong independent risk factors for lactic acidosis on ICU admission in patients undergoing cardiac surgery [17,42,43,44]. While mildly elevated lactate levels after cardiac surgery are frequently transient and usually considered benign [41,45], studies have consistently demonstrated that elevated lactate levels > 4 mmol/L are a prognostic marker for worse outcomes, including mortality after cardiac surgery [13,14,17,41]. Nevertheless, cluster 1 patients in our study had the lowest incidence of persistent lactic acidosis, and lowest in-hospital and 90-day mortality risks among the three clusters.

Patients in cluster 2 were the youngest and had the highest baseline eGFR among the three clusters. Cluster 2 consisted of surgical patients, especially those admitted to trauma surgical ICU (TSICU) following trauma. They had the highest blood pressure values, least severe anemia, and lowest vasopressor requirement among the three clusters. While cluster 2 patients had the highest hemoglobin among the three clusters, this could have been due to blood transfusion administered prior to ICU admission for trauma and surgical patients [46,47,48,49]. In addition, cluster 2 had the lowest serum potassium, magnesium, and phosphate levels. Average lactate levels in the cluster 2 were 5.6 mmol/L, which is comparable to cluster 1. Lactic acidosis in patients after trauma often occurs due to the metabolic response from an oxygen supply-demand mismatch in the setting of hypoxia, hemorrhage, and anaerobic metabolism [46,47,48,49]. Lactate level has been shown to be a prognostic biomarker in trauma, even in patients without hypotension [9,10,47] and elevated blood lactate levels (>4 mmol/L) among trauma and surgical patients admitted to the ICU are closely correlated with worse patient outcomes and a reduced chance of survival [50,51,52,53,54,55,56,57,58,59,60]. Our study demonstrated that 9.8% of patients in cluster 2 developed persistent lactic acidosis, which is comparable to cluster 1. Compared to cluster 1, patients in cluster 2 had increased in-hospital and 90-day mortality.

Among all clusters, cluster 3 had the highest degree of lactic acidosis. They also had the lowest arterial pH, pO_2_, and HCO_3_ levels. Moreover, cluster 3 patients had the highest prevalence of liver disease, coagulopathy, fluid and electrolyte disorders, and metastatic cancer. More than half of these patients were admitted to the MICU. Cluster 3 patients also had the highest Charlson and SAPs II scores, signifying high comorbidity burden and severity of acute illness. On ICU admission, they had the lowest blood pressure but the highest incidence of positive blood culture, acute kidney injury, and hyperphosphatemia. Thus, lactic acidosis in cluster 3 patients likely occurred in the setting of tissue hypoperfusion due to shock. Additionally, liver disease and metastatic cancer may have contributed to a type B lactic acidosis [61,62,63]. While diabetes was not a main feature that differentiated the three clusters, it was noted to be more prevalent in cluster 3 than other clusters, and metformin is an uncommon but important cause of type B lactic acidosis in the ICU [20,21]. In addition, the higher prevalence of liver disease and greater rate of AKI in this cluster could also lead to a reduction of lactate clearance. Altogether, cluster 3 patients had the highest incidence of persistent lactic acidosis in 48 h, in-hospital mortality, and 90-day mortality among the three clusters.

The strengths of our study include innovative findings via an unbiased and easily reproducible unsupervised ML consensus clustering approach derived from a large sample population of ICU patients with lactic acidosis. Nevertheless, there are several important limitations. First, we did not have information on blood transfusion prior to ICU admission, and thus cluster 2 with its association with the highest hemoglobin could have been affected by blood transfusions that commonly occur in trauma patients. Furthermore, data on medications prior to ICU admission (such as metformin or vasopressors) were lacking. Thus, we could not investigate whether metformin or other known causes of drug-induced lactic acidosis may have played an important role in the clustering approach of our study. Future studies are needed to assess whether the incorporation of these variables could have improved the discriminatory ability of the clusters we identified. Lastly, consensus clustering was performed on ICU admission and did not include clinical data before or during ICU stay, which could affect ICU-related outcomes. Nevertheless, we only included data that were readily available at the time of ICU admission as this would mimic the phenotype that clinicians would initially be provided with. By this method, we successfully identified distinct clusters of lactic acidosis on ICU admission that were associated with unique clinical outcomes, including persistent lactic acidosis, in-hospital mortality, and 90-day mortality.

## 5. Conclusions

In conclusion, we present an unsupervised ML consensus clustering analysis of critically ill patients with lactic acidosis on ICU admission. We discovered three distinct phenotypes of lactic acidosis on ICU admission with unique characteristics and hospital outcomes. While our findings provide a better understanding of the associated different outcomes for distinct subtypes of patients with lactic acidosis on ICU admission, future studies are needed to further investigate the impact of ML application clustering analysis to the care of ICU patients with lactic acidosis.

## Figures and Tables

**Table 1:** Clinical characteristics at ICU admission according to clusters of lactic acidosis.

Characteristics	Overall	Cluster 1	Cluster 2	Cluster 3	*p*-Value
	(*n* = 1919)	(*n* = 554)	(*n* = 815)	(*n* = 550)	
Age (years)	61.8 ± 17.1	68.9 ± 12.4	54.7 ± 18.4	65.0 ± 15.1	<0.001
Male sex	1118 (58)	295 (53)	498 (61)	325 (59)	0.01
Race					
− White	1560 (81)	487 (88)	647 (79)	426 (77)	<0.001
− Black	152 (8)	20 (4)	66 (8)	66 (12)	
− Hispanic	79 (4)	14 (2)	46 (6)	19 (4)	
− Other	128 (7)	33 (6)	56 (7)	39 (7)	
ICU type					
− Cardiac ICU	206 (11)	20 (3)	90 (11)	96 (17)	<0.001
− Cardiac surgery ICU	467 (24)	392 (71)	52 (7)	23 (4)	
− Medical ICU	605 (32)	28 (5)	255 (31)	322 (59)	
− Surgical ICU	295 (15)	76 (14)	148 (18)	71 (13)	
− Trauma/surgical ICU	346 (18)	38 (7)	270 (33)	38 (7)	
Elixhauser Comorbidities					
− Congestive heart failure	456 (24)	185 (33)	118 (14)	153 (28)	<0.001
− Valvular disease	352 (18)	263 (47)	41 (5)	48 (9)	<0.001
− Pulmonary circulation disorders	133 (7)	49 (9)	36 (4)	48 (9)	0.001
− Peripheral vascular disease	286 (15)	182 (33)	49 (6)	55 (10)	<0.001
− Hypertension	884 (46)	345 (62)	286 (35)	253 (46)	<0.001
− Paralysis	55 (3)	19 (3)	26 (3)	10 (2)	0.21
− Neurologic disorders	174 (9)	29 (5)	100 (12)	45 (8)	<0.001
− Chronic pulmonary disease	266 (14)	87 (16)	108 (13)	71 (13)	0.33
− Uncomplicated diabetes	385 (20)	115 (21)	133 (16)	137 (25)	<0.001
− Complicated diabetes	73 (4)	29 (5)	10 (1)	34 (6)	<0.001
− Hypothyroidism	134 (7)	58 (10)	33 (4)	43 (8)	<0.001
− Liver disease	291 (15)	64 (12)	72 (9)	155 (28)	<0.001
− Peptic ulcer	1 (0.05)	0 (0)	1 (0.1)	0 (0)	0.51
− AIDS/HIV	27 (1)	4 (1)	9 (1)	14 (3)	0.02
− Lymphoma	52 (3)	7 (1)	18 (2)	27 (5)	<0.001
− Metastatic cancer	136 (7)	17 (3)	39 (5)	80 (15)	<0.001
− Solid tumor	128 (7)	34 (6)	53 (7)	41 (7)	0.66
− Rheumatoid arthritis	41 (2)	19 (3)	10 (1)	12 (2)	0.02
− Coagulopathy	500 (26)	138 (25)	123 (15)	239 (43)	<0.001
− Obesity	97 (5)	39 (7)	36 (4)	22 (4)	0.04
− Weight loss	68 (4)	14 (3)	20 (2)	34 (6)	<0.001
− Fluid and electrolyte disorders	843 (44)	158 (29)	314 (39)	371 (67)	<0.001
− Blood loss anemia	36 (2)	11 (2)	10 (1)	15 (3)	0.13
− Deficiency anemia	275 (14)	79 (14)	105 (13)	91 (17)	0.17
− Alcohol abuse	199 (10)	24 (4)	110 (13)	65 (12)	<0.001
− Drug abuse	70 (4)	9 (2)	45 (6)	16 (3)	<0.001
− Psychosis	71 (4)	10 (2)	40 (5)	21 (4)	0.01
− Depression	104 (5)	25 (5)	53 (7)	26 (5)	0.20
Charlson comorbidity score	4.4 ± 2.7	4.9 ± 2.3	3.0 ± 2.4	5.8 ± 2.7	<0.001
Vital signs					
− Temperature (F)	97.2 ± 2.2	96.7 ± 1.9	97.7 ± 2.1	97.0 ± 2.4	<0.001
− Heart rate (per minute)	97 ± 21	87 ± 15	100 ± 22	102 ± 23	<0.001
− Systolic blood pressure (mmHg)	117 ± 26	116 ± 21	126 ± 26	105 ± 24	<0.001
− Diastolic blood pressure (mmHg)	62 ± 15	58 ± 11	69 ± 15	56 ± 14	<0.001
− Mean blood pressure (mmHg)	81 ± 21	78 ± 14	89 ± 22	74 ± 21	<0.001
− Respiratory rate (per minute)	17 ± 9	12 ± 7	18 ± 8	22 ± 9	<0.001
− Oxygen saturation (%)	97 ± 5	98 ± 3	97 ± 4	95 ± 6	<0.001
− Glasgow coma score	8 ± 5	5 ± 4	9 ± 5	10 ± 5	<0.001
Vasopressor use	1230 (64)	446 (80)	361 (44)	423 (77)	<0.001
Ventilator use	1608 (84)	540 (97)	640 (79)	428 (78)	<0.001
Any renal replacement therapies	54 (3)	11 (2)	14 (2)	29 (5)	<0.001
− Hemodialysis	35 (2)	7 (1)	9 (1)	19 (3)	0.003
− CRRT	22 (1)	6 (1)	5 (1)	11 (2)	0.06
SAPS II score	61 ± 20	63 ± 14	52 ± 18	73 ± 20	<0.001
Acute kidney injury	1401 (73)	422 (76)	494 (61)	485 (88)	<0.001
Laboratory data					
− BUN (mg/dL)	27 ± 21	20 ± 11	19 ± 11	46 ± 27	<0.001
− eGFR (mL/min/1.73 m^2^)	68 ± 31	69 ± 23	80 ± 29	50 ± 32	<0.001
− Sodium (mEq/L)	138 ± 5	137 ± 4	139 ± 5	138 ± 6	<0.001
− Potassium (mEq/L)	4.4 ± 0.9	4.6 ± 0.9	4.0 ± 0.7	4.6 ± 1.0	<0.001
− Chloride (mEq/L)	106 ± 7	108 ± 5	107 ± 6	104 ± 8	<0.001
− Bicarbonate (mEq/L)	20 ± 5	23 ± 4	20 ± 4	16 ± 5	<0.001
− Anion gap (mEq/L)	18 ± 6	14 ± 4	17 ± 4	22 ± 6	<0.001
− Total calcium (mg/dL)	8.2 ± 1.2	8.7 ± 1.2	8.0 ± 1.1	7.9 ± 1.1	<0.001
− Ionized calcium (mmol/L)	1.1 ± 0.2	1.2 ± 0.2	1.1 ± 0.1	1.0 ± 0.1	<0.001
− Phosphate (mg/dL)	4.1 ± 1.8	3.8 ± 1.3	3.5 ± 1.4	5.3 ± 2.1	<0.001
− Magnesium (mg/dL)	1.9 ± 0.5	2.1 ± 0.6	1.7 ± 0.4	2.1 ± 0.5	<0.001
− Lactate (mmol/L)	6.2 ± 2.6	5.7 ± 1.9	5.6 ± 1.7	7.7 ± 3.4	<0.001
− Glucose (mg/dL)	179 ± 89	170 ± 63	185 ± 87	181 ± 111	0.009
− Hemoglobin (g/dL)	10.6 ± 2.3	9.1 ± 1.8	11.9 ± 2.1	10.1 ± 2.1	<0.001
− WBC (10^9^ cells/L)	14.1 ± 8.3	12.4 ± 6.4	14.4 ± 8.1	15.4 ± 10.0	<0.001
− Platelet (10^9^ cells/L)	170 ± 103	146 ± 68	208 ± 103	172 ± 120	<0.001
− pH	7.31 ± 0.12	7.36 ± 0.10	7.32 ± 0.10	7.26 ± 0.13	<0.001
− pCO_2_ (mmHg)	39 ± 11	41 ± 9	40 ± 11	36 ± 2	<0.001
− pO_2_ (mmHg)	209 ± 133	309 ± 117	180 ± 118	151 ± 113	<0.001
− PT (second)	18 ± 6	17 ± 4	16 ± 4	22 ± 9	<0.001
− INR	1.8 ± 1.0	1.6 ± 0.5	1.6 ± 0.6	2.5 ± 1.6	<0.001
− PTT (second)	49 ± 30	54 ± 31	40 ± 24	56 ± 34	<0.001
Culture data, *n* (%)					
− Positive blood culture	197 (10)	7 (1)	76 (9)	114 (21)	<0.001
− Positive urine culture	284 (15)	32 (6)	138 (17)	114 (21)	<0.001
− Positive sputum culture	205 (11)	23 (4)	76 (9)	106 (19)	<0.001

Continuous variables were reported as mean ± standard deviation, categorical variables as count (percentage). Abbreviations: AIDS, acquired immune deficiency syndrome; BUN, blood urea nitrogen; CRRT, continuous renal replacement therapy; eGFR, estimated glomerular filtration rate; HIV, human immunodeficiency virus; ICU, intensive care unit; INR, international normalized ratio; pH, potential of hydrogen; pCO_2_, partial pressure of carbon dioxide; pO_2_, partial pressure of oxygen; PT, prothrombin time; PTT, partial thromboplastin time; SAPS II score, simplified acute physiology score II; WBC, white blood cell.

**Table 2:** Outcomes according to clusters of lactic acidosis.

Cluster	Persistent Lactic Acidosis		Hospital Mortality		90-Day Mortality	
	%	OR (95% CI)	%	OR (95% CI)	%	HR (95% CI)
Cluster 1	9.2%	1 (ref)	14.6%	1 (ref)	19.9%	1 (ref)
Cluster 2	9.8%	1.08 (0.73–1.59)	20.9%	1.54 (1.15–2.06)	25.9%	1.38 (1.10–1.74)
Cluster 3	40.0%	6.59 (4.62–9.39)	58.2%	8.12 (6.08–10.86)	66.6%	5.06 (4.09–6.27)

## Data Availability

Data is available upon reasonable request to the corresponding author.

## Supplementary Materials

The following are available online at https://www.mdpi.com/article/10.3390/jpm11111132/s1, Figures S1–S9: Consensus matrix heat map; Supplementary method: detailed cluster derivation.
