# Supplementary material for: Machine Learning Consensus Clustering Approach for Patients with Lactic Acidosis in Intensive Care Units
Source: J Pers Med. 2021 Nov 2;11(11):1132. doi: 10.3390/jpm11111132 (PMC8623582; doi:10.3390/jpm11111132)
Supplement: Supplementary file 1 [file jpm-11-01132-s001.zip › jpm-1410584-supplementary.pdf]

Cluster derivation

We applied an unsupervised ML approach to develop clinical phenotypes of ICU patients with lactic acidosis by conducting unsupervised consensus clustering.[1] We performed consensus clustering analysis on the whole study population. We initially assessed the distribution and missingness in phenotyping variables. We included only variables that had <10% missing values. Subsequently, missing data were imputed through multiple imputation using Random Forest,[2, 3] and Non-normal data were z-score normalized. Random Forest imputation is a nonparametric algorithm that accommodates nonlinearities and interactions and does not require the specification of a particular parametric model.[4] This approach generated single-point estimates by random draws from independent normal distributions centered on conditional means predicted by random Forest. Random Forest applies bootstrap aggregation of multiple regression trees to reduce the risk of overfitting, and combines estimates from many trees.[5] We subsequently applied clustering using the consensus cluster algorithm. The algorithm begins by subsampling a proportion of items and a proportion of features from a data matrix. Each subsample is then partitioned into up to groups (k) by a user-specified clustering algorithm. This process is repeated for a specified number of times. Pairwise consensus values, defined as 'the proportion of clustering runs in which two items are grouped together', are calculated and stored in a consensus matrix (CM) for each cluster. Clustering settings used were as follows: maximum number of clusters, 10; number of iterations, 100; subsampling fraction, 0.8; clustering algorithm, K-means; Euclidean distance).[1] The number of potential clusters ranges from 2 to 10, to avoid producing an excessive number of clusters that would not be clinical useful. Pairwise consensus values, defined as 'the proportion of clustering runs in which two items are [grouped] together[1], are calculated and stored in a CM for each k. Then for each k, a final agglomerative hierarchical consensus clustering using distance of 1−consensus values is completed and pruned to k groups, which are called consensus clusters.

The clustering algorithm is to maximize the potential number of clusters while maintaining high cluster consensus. The optimal number of clusters was determined by examining the CM heat map, cumulative distribution function, cluster-consensus plots with the within-cluster consensus scores, and the proportion of ambiguously clustered pairs (PAC).[6, 7] The within-cluster consensus score, ranging between 0 and 1, is defined as the average consensus value for all pairs of individuals belonging to the same cluster.[7] A value closer to one indicates better cluster stability.[7] PAC, ranging between 0 and 1, is calculated as the proportion of all sample pairs with consensus values falling within the predetermined boundaries.[6] A value closer to zero indicates

better cluster stability.[6] We calculated the PAC using two criteria: the strict criteria had the predetermined boundary of (0, 1), where a pair of individuals who had a consensus value greater than 0 or less than 1 was considered ambiguously clustered, and the relaxed criteria had the predetermined boundary of (0.1, 0.9), where a pair of individuals who had a consensus value greater than 0.1 or less than 0.9 was considered ambiguously clustered; i.e., a pair was unambiguously clustered if the two individuals were clustered in the same cluster for either more than 90% of the time or less than 10% of the time (meaning they were not in the same cluster for more than 90% of the time).[6]

Calculation of the standardized difference of each parameter used the cutoff of ±0.3 to show subgroup features with the key features for each cluster. All cluster derivation analyses were performed using R, version 4.0.3 (RStudio, Inc., Boston, MA; http://www.rstudio.com/), with the packages of ConsensusClusterPlus (version 1.46.0)[7]. Missing data were imputed using the Random Forest method for each study cohort with the missForest package.[5] All analyses were two-tailed, and P value < .05 was considered statistically significant.

Supplementary Figure S1

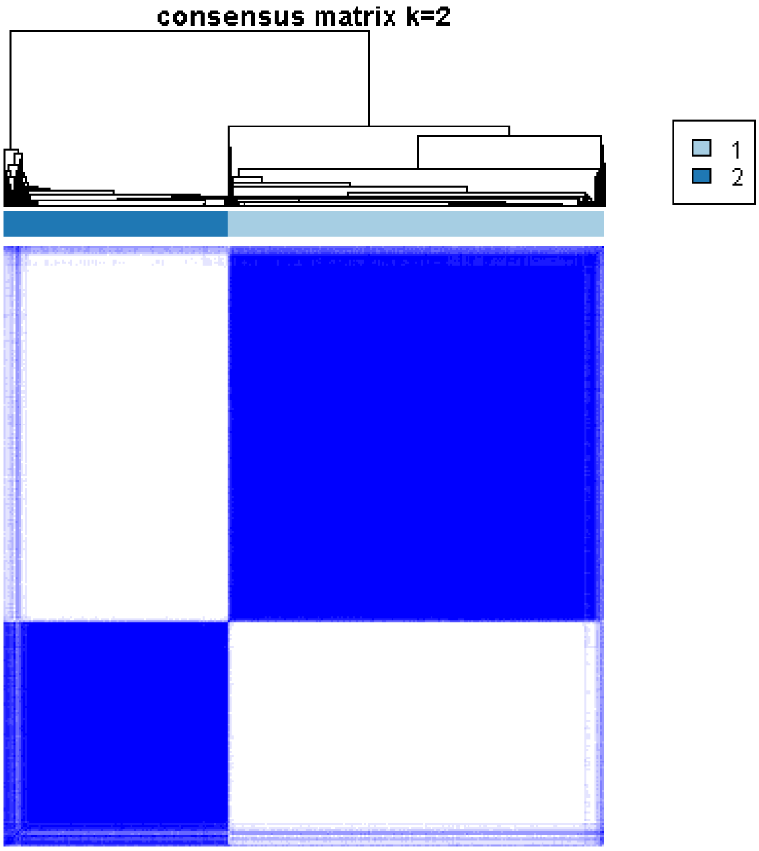

Supplementary Figure S2

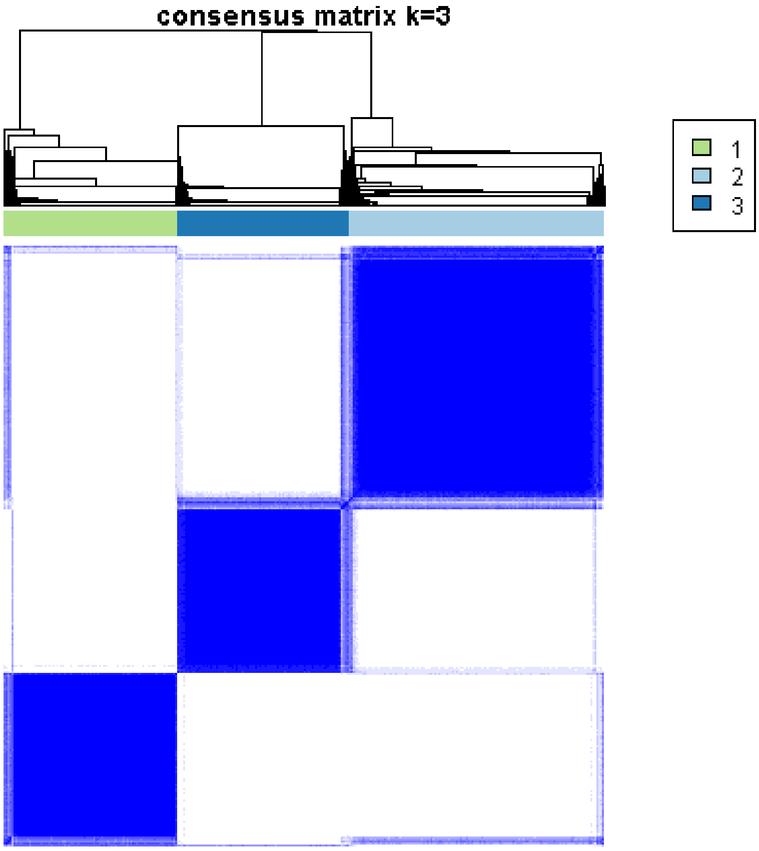

Supplementary Figure S3

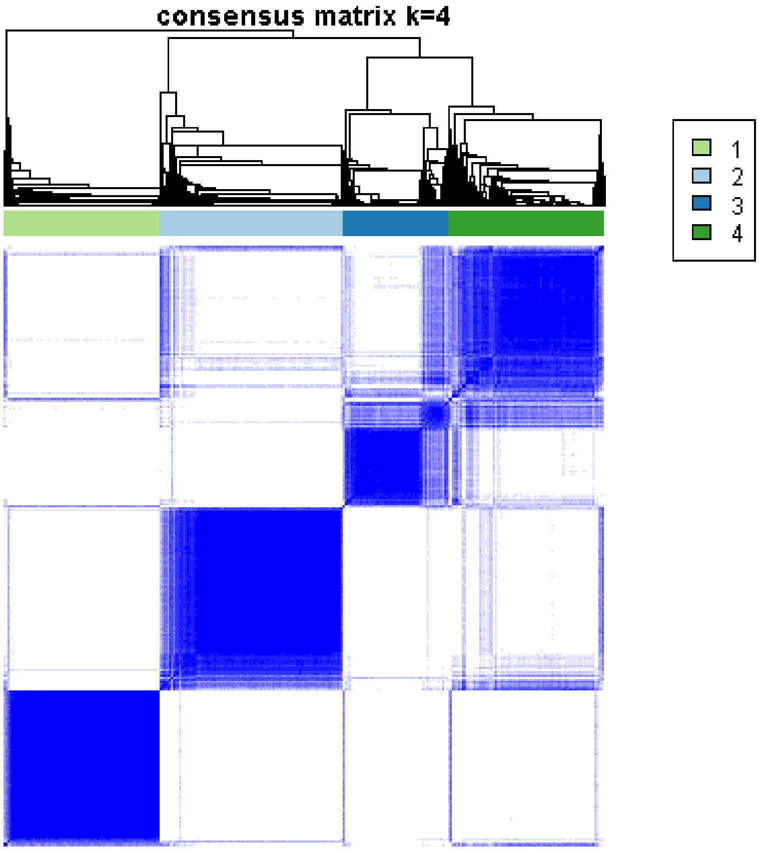

Supplementary Figure S4

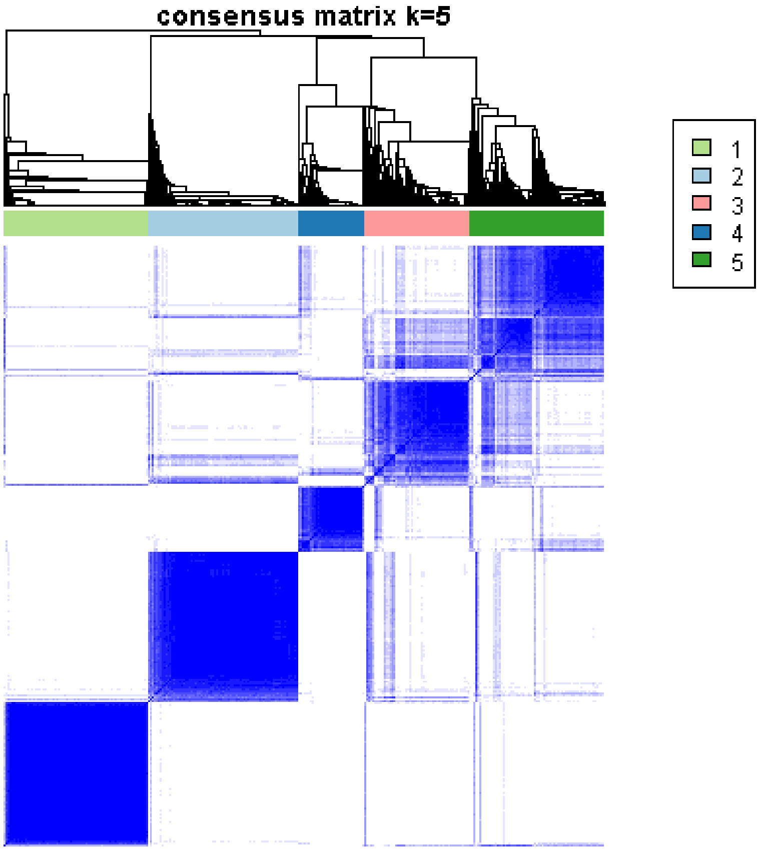

Supplementary Figure S5

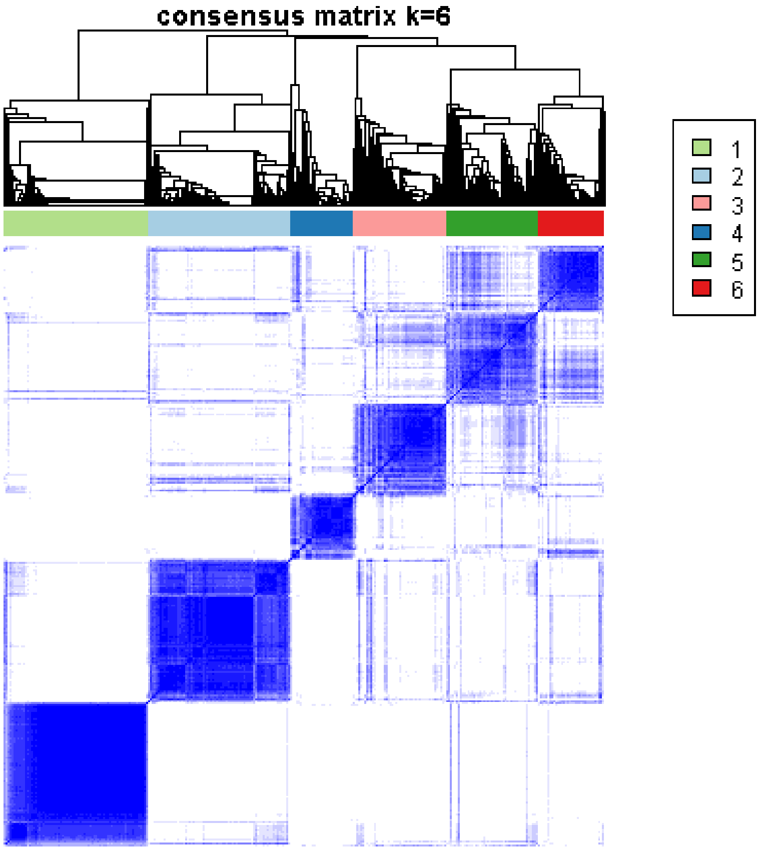

Supplementary Figure S6

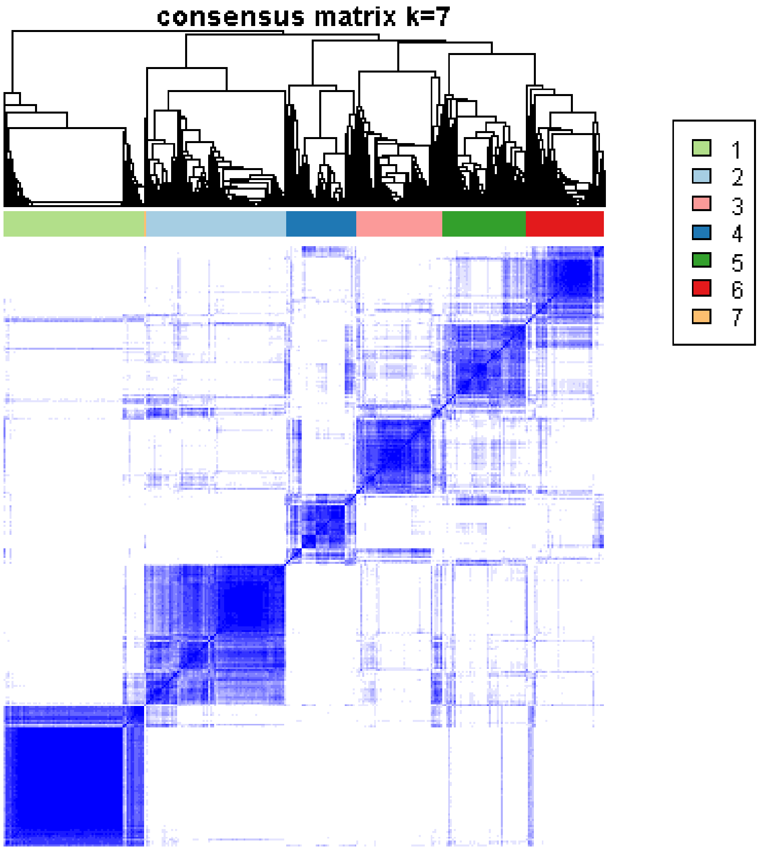

Supplementary Figure S7

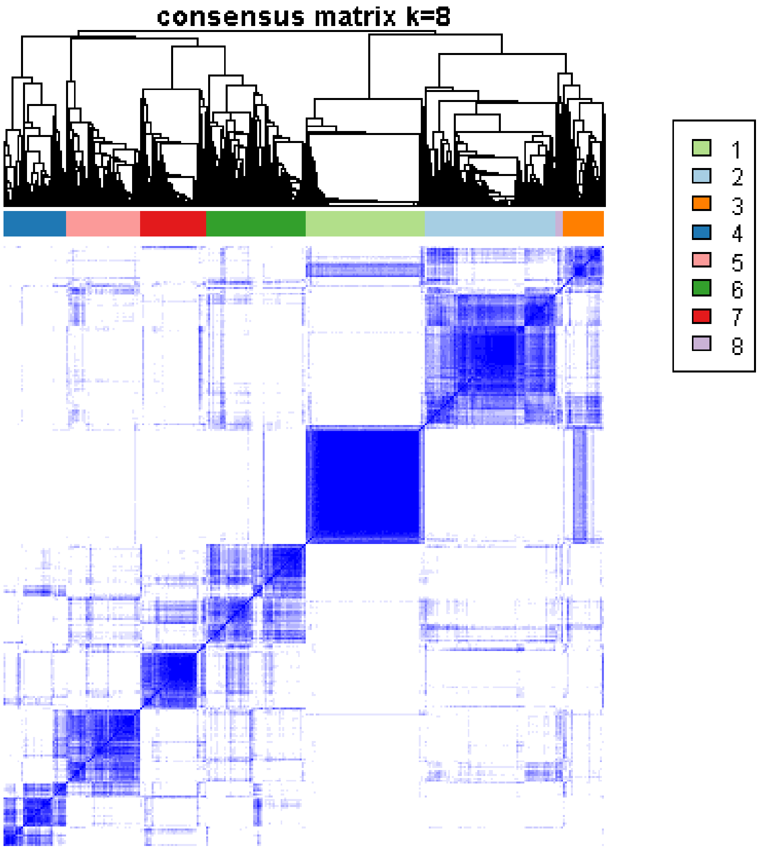

Supplementary Figure S8

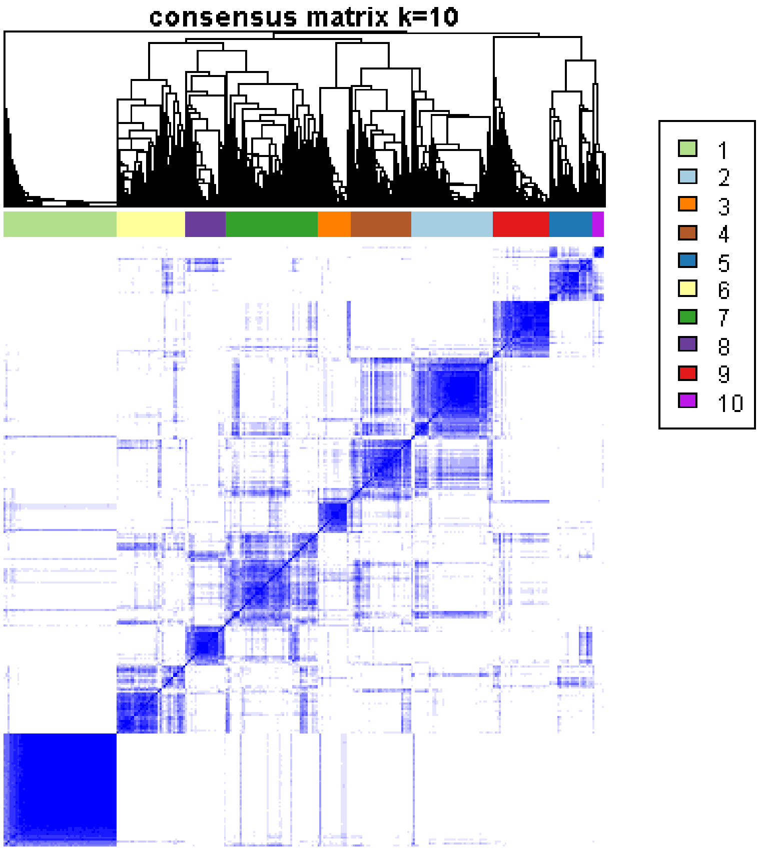

Supplementary Figure S9

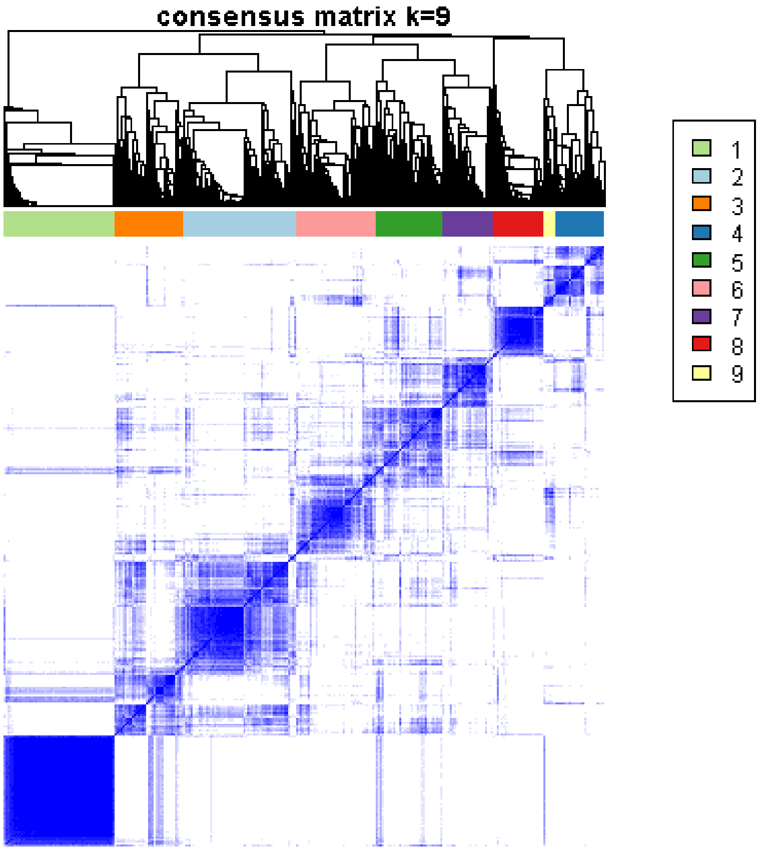

References

1 Monti S, Tamayo P, Mesirov J, Golub T. Consensus clustering: a resampling-based method for class discovery and visualization of gene expression microarray data. Machine learning. 2003: 91
2 Pantanowitz A, Marwala T. Missing data imputation through the use of the random forest algorithm. Advances in Computational Intelligence: Springer; 2009. p. 53-62.
3 Tang F, Ishwaran H. Random forest missing data algorithms. Statistical Analysis and Data Mining: The ASA Data Science Journal. 2017: 363
4 Shah AD, Bartlett JW, Carpenter J, Nicholas O, Hemingway H. Comparison of random forest and parametric imputation models for imputing missing data using MICE: a CALIBER study. American journal of epidemiology. 2014: 764
5 Stekhoven DJ, Bühlmann P. MissForest—non-parametric missing value imputation for mixed-type data. Bioinformatics. 2012: 112
6 Șenbabaoğlu Y, Michailidis G, Li JZ. Critical limitations of consensus clustering in class discovery. Sci Rep. 2014: 6207 [PMID: 25158761 10.1038/srep06207: 10.1038/srep06207]
7 Wilkerson MD, Hayes DN. ConsensusClusterPlus: a class discovery tool with confidence assessments and item tracking. Bioinformatics. 2010: 1572
